# Mitochondrial Adaptations in Aging Skeletal Muscle: Implications for Resistance Exercise Training to Treat Sarcopenia

**DOI:** 10.3390/life14080962

**Published:** 2024-07-31

**Authors:** Ilyoung Jeong, Eun-Jeong Cho, Jang-Soo Yook, Youngju Choi, Dong-Ho Park, Ju-Hee Kang, Seok-Hun Lee, Dae-Yun Seo, Su-Jeen Jung, Hyo-Bum Kwak

**Affiliations:** 1 Program in Biomedical Science & Engineering, Department of Biomedical Science, Inha University, Incheon 22212, Republic of Korea; 10hello@inha.edu (I.J.); cejeong97@naver.com (E.-J.C.); dparkosu@inha.ac.kr (D.-H.P.); johykang@inha.ac.kr (J.-H.K.); 2 Institute of Sports and Arts Convergence, Inha University, Incheon 22212, Republic of Korea; yookjs@inha.ac.kr (J.-S.Y.); choiyoungju0323@gmail.com (Y.C.); 3 Institute of Specialized Teaching and Research, Inha University, Incheon 22212, Republic of Korea; 4 Department of Kinesiology, Inha University, Incheon 22212, Republic of Korea; 5 Department of Pharmacology, College of Medicine, Inha University, Incheon 22212, Republic of Korea; 6 Combat Institute of Australia, Leederville, WA 6007, Australia; seokhun.lee@combataus.com.au; 7 Basic Research Laboratory, Department of Physiology, College of Medicine, Smart Marine Therapeutic Center, Cardiovascular and Metabolic Disease Core Research Support Center, Inje University, Busan 47392, Republic of Korea; 8 Department of Leisure Sports, Seoil University, Seoul 02192, Republic of Korea

**Keywords:** aging, resistance exercise training, mitochondria, sarcopenia, skeletal muscle

## Abstract

Sarcopenia, the age-related decline in muscle mass and function, poses a significant health challenge as the global population ages. Mitochondrial dysfunction is a key factor in sarcopenia, as evidenced by the role of mitochondrial reactive oxygen species (mtROS) in mitochondrial biogenesis and dynamics, as well as mitophagy. Resistance exercise training (RET) is a well-established intervention for sarcopenia; however, its effects on the mitochondria in aging skeletal muscles remain unclear. This review aims to elucidate the relationship between mitochondrial dynamics and sarcopenia, with a specific focus on the implications of RET. Although aerobic exercise training (AET) has traditionally been viewed as more effective for mitochondrial enhancement, emerging evidence suggests that RET may also confer beneficial effects. Here, we highlight the potential of RET to modulate mtROS, drive mitochondrial biogenesis, optimize mitochondrial dynamics, and promote mitophagy in aging skeletal muscles. Understanding this interplay offers insights for combating sarcopenia and preserving skeletal muscle health in aging individuals.

## 1. Introduction

A decline in physical fitness naturally occurs as individuals age, leading to increased vulnerability to both physical and psychological impairments [1]. This is reflected in an increased prevalence of chronic health issues. A key feature of aging is the progressive loss of muscle mass, strength, and function, known as sarcopenia [2,3].

Loss of muscle mass in older adults is associated not only with falls and fractures but also with an increased risk of chronic diseases such as diabetes, hypertension, heart disease, and cancer [2,4,5]. The etiology of sarcopenia involves a range of factors and pathways, ranging from environmental influences such as physical inactivity and poor diet to cellular changes such as reduced satellite cell counts, activation of apoptotic pathways, and mitochondrial dysfunction [2]. Emerging evidence indicates that mitochondrial dysfunction and the activation of apoptotic pathways play pivotal roles in the development of age-related sarcopenia [6].

Mitochondria serve as the powerhouses of cells, responsible for producing adenosine triphosphate (ATP), and thus play a critical role in central metabolic pathways and cellular functions, ultimately impacting the overall health of the individual. Deficiencies in these processes can lead to various diseases, such as Alzheimer’s and cancer [7]. Furthermore, the mitochondria play a crucial role in regulating the metabolic function of skeletal muscles, and mitochondrial dysfunction directly affects normal skeletal muscle functioning [8]. In older adults with sarcopenia, skeletal muscle mitochondria demonstrate a characteristic decline in the expression and activity of mitochondrial respiratory complexes, accompanied by suppressed oxidative phosphorylation and downregulation of genes responsible for mitochondrial protein quality control [9].

Exercise provides numerous benefits, including improved cardiovascular health, enhanced skeletal muscle and bone health, chronic disease prevention, longevity, and improved immune function in aging individuals with sarcopenia [10]. Resistance exercise training (RET) and aerobic exercise training (AET) are two distinct exercise modalities with well-documented health benefits in aging patients with sarcopenia. Typically, the adaptation of skeletal muscles to exercise is often viewed through a dichotomous lens, where AET induces increased mitochondrial adaptations, whereas RET leads to myofibrillar adaptations through mechanical tension [11]. AET improves endurance, oxidative capacity, mitochondrial content, and function [12,13]. A systematic review and meta-analysis of 20 randomized controlled trials (RCTs) demonstrated that regular AET significantly reduces oxidative stress markers and increases antioxidant levels in older adults [14]. Furthermore, Konopka et al. examined mitochondrial quality control in young and older men. They found that 12 weeks of AET promoted mitochondrial biogenesis, as evidenced by increased levels of related proteins and elevated metabolic enzyme activity in both age groups [15]. Interestingly, the authors also reported an increase in markers of both mitochondrial fusion and fission following AET, regardless of age [15]. However, the effects of AET on mitophagy and selective removal of damaged mitochondria remain a topic of debate [16]. Researchers found in one study of an aged animal model of sarcopenia a decrease in mitophagic flux, potentially due to improved overall mitochondrial quality following endurance exercise training [17]; another report contradicted this finding. These results suggest that 4 to 5 weeks of voluntary wheel-running exercise promotes elevated basal mitophagy in trained skeletal muscles, potentially reflecting a heightened rate of mitochondrial turnover in skeletal muscle [18]. In contrast, RET research has primarily focused on adaptations related to the enhancement of skeletal muscle hypertrophy and strength [19]. The impact of RET on mitochondrial adaptation in aging skeletal muscles remains inconclusive. Traditionally viewed as less effective than endurance exercise in improving mitochondrial function, recent studies have reported enhanced markers of mitochondrial content and function following RET in both young [20,21] and old populations [22,23,24]. Further research is required to determine how much RET contributes to mitochondrial adaptation.

In this review, we discuss the underappreciated role of RET in aging skeletal muscle mitochondria, offering a new perspective on its therapeutic benefits in combating age-related sarcopenia. We summarize the effects of RET on mitochondria in aging skeletal muscles and focus on mitochondrial reactive oxygen species (mtROS), biogenesis, dynamics, and mitophagy, exploring the potential underlying mechanisms.

## 2. Role of Mitochondria in Sarcopenia

Skeletal muscle loss is influenced by several factors and pathways, including chronic inflammation, reduced growth hormone signaling, and inadequate protein intake [25,26,27]. Furthermore, sarcopenia entails a complex interplay of factors, such as mitochondrial dysfunction, leading to impaired skeletal muscle fiber energy production, malfunctioning muscle satellite cell function, and neurological impairments affecting motor unit recruitment and innervation [28]. Recent research suggests that changes in mitochondrial biogenesis, dynamics, function, and structure may be the primary factors influencing skeletal muscle quality and performance [29]. Understanding how these factors affect skeletal muscle metabolism is key to developing effective strategies for preventing and treating sarcopenia and, ultimately, promoting healthy aging.

### 2.1. Mitochondrial Reactive Oxygen Species and Mitochondrial Respiration

The overexpression of mtROS causes oxidative stress and is a major contributor to skeletal muscle weakness and loss during sarcopenia. mtROS are produced as a by-product of the electron transport chain (ETC). The excessive production of mtROS leads to loss of muscle mass and skeletal muscle strength, protein damage, mitochondrial dysfunction, and mitochondrial DNA (mtDNA) damage [30]. Skeletal muscle fibers experiencing higher levels of oxidative stress appear to have a greater prevalence of mtDNA deletions and mutations. The mtDNA genome is particularly vulnerable to mutational damage because of its proximity to the electron transport system, a major source of free radicals [31,32]. The accumulation of unrepaired mtDNA damage can exacerbate mitochondrial dysfunction by promoting increased mtROS production [33]. mtDNA mutations can impair mitochondrial respiration by damaging ETC complexes, leading to the presence of defective subunits within the ETC, which disrupts oxidative phosphorylation, reduces ATP synthesis, and elevates reactive oxygen species (ROS) generation [34,35,36,37,38]. Consequently, mitochondrial respiration, the cellular process that utilizes oxygen to generate ATP, is further impaired by mtDNA mutations [37,39].

Studies suggest elevated mtROS and impaired skeletal muscle mitochondrial respiration are associated with age-related sarcopenia [40,41]. For example, Grevendonk et al. investigated the impact of aging on mitochondrial function in older and young individuals maintaining similar levels of habitual physical activity [42]. To assess skeletal muscle mitochondrial respiration, the researchers employed high-resolution respirometry with permeabilized skeletal muscle fibers. They found approximately 15% lower ADP-stimulated mitochondrial respiration rate in older adults relative to young adults. However, no significant difference in mitochondrial content, assessed by mitochondrial oxidative phosphorylation (OXPHOS) protein levels, was observed between the groups [42]. Similarly, Joseph et al. observed a more specific association between low physical function and mitochondrial dysfunction in aging muscle [43]. They reported a decline in mitochondrial respiration, potentially leading to higher free radical formation and subsequent muscle damage, in elderly individuals with low physical function [43]. Furthermore, González-Blanco et al. reported that severely functional-dependent patients with extreme sarcopenia have lower expression of the main complexes of the ETC [44]. This finding suggests a decline in mitochondrial function and a reduced ability to produce energy (ATP) within muscle cells. Fan et al. reported higher levels of circulating cell-free mitochondrial DNA (ccf-mtDNA) in older adults with sarcopenia [45]. ccf-mtDNA, a short segment of mtDNA derived from cells, is released into systemic circulation in response to cellular injury or stress [46]. Elevated ccf-mtDNA levels may indicate mitochondrial damage due to abnormal mitochondrial leakage [45]. Damage to mtDNA caused by ROS is thought to initiate a cycle of increasing oxidative stress within the mitochondria [47].

### 2.2. Mitochondrial Biogenesis

Reduced mitochondrial biogenesis, which involves not only generating new mitochondria but also increasing their size and mass, is a key factor in the loss of skeletal muscle quality and the development of sarcopenia [29,48,49]. Mitochondrial biogenesis is a complex process that involves building proteins encoded by mtDNA, importing nuclear-encoded mitochondrial proteins, and replicating mtDNA [50]. Studies have shown that peroxisome proliferator-activated receptor-gamma coactivator 1 alpha (PGC-1α) plays a pivotal role in mitochondrial biogenesis by regulating the transcriptional machinery responsible for increasing mitochondrial mass [51,52]. The levels of PGC-1α protein and mRNA decline with age, and this decrease is associated with skeletal muscle weakness and poor exercise performance in older adults [49]. Joseph et al. demonstrated that lower physical function in older adults correlates with weaker mitochondrial function and reduced PGC-1α protein levels compared with both younger adults and high-functioning older adults [43]. Cannavino et al. reported that mice genetically modified to have high levels of PGC-1α maintained skeletal muscle mass even when the hindlimb was unloaded, potentially by suppressing autophagy and proteasome degradation pathways that break down skeletal muscle proteins [53]. However, a recent study investigating hindlimb unloading in mice overexpressing PGC-1α did not observe a protective effect against disuse atrophy with respect to muscle mass or cross-sectional area [54]. This discordance in results may be attributable to differences in the age of the mice used in the studies [54]. Aging in skeletal muscles is associated with altered mitochondrial biogenesis pathways, which may contribute to skeletal muscle loss and reduced physical function commonly observed in older adults [43].

### 2.3. Mitochondrial Dynamics

Mitochondrial dysfunction, caused by altered dynamics and morphology, is a major contributor to the decline in skeletal muscle function observed in sarcopenia [29,55]. Mitochondria are dynamic organelles that constantly undergo fusion and fission, which are important for maintaining mitochondrial function. Mitochondrial fusion is the merging of two mitochondria at the interfaces of their outer and inner membranes. In contrast, mitochondrial fission refers to the process by which a mitochondrion is divided into two separate mitochondria [56]. The constant interplay between fusion and fission determines the size, shape, and number of mitochondria in a cell [57]. In healthy cells, these processes are balanced to maintain the mitochondrial morphology. Mitochondrial fusion occurs in a two-step process. The fusion process involves the facilitation of the outer mitochondrial membrane by Mitofusin 1 (Mfn1) and Mitofusin 2 (Mfn2), followed by the mediation of inner mitochondrial membrane fusion by the Optic Atrophy 1 protein (OPA1) [8]. Mitochondrial fission involves dynamin-related protein 1 (Drp1), which forms constricting rings around the organelle and is recruited by mitochondrial Fission-1 protein (Fis1) to the outer membrane [57]. The results of recent studies suggest that disruptions in the balance of mitochondrial dynamics can impair mitochondrial function and health in skeletal muscles [29,56,57]. This imbalance is associated with senescence and muscle atrophy. For example, Sebastián et al. reported that Mfn2 deletion leads to the accumulation of damaged mitochondria, increased production of harmful molecules, impaired skeletal muscle function, reduced energy production, and general weakness [58]. In addition, Favaro et al. showed that the absence of Drp1 leads to abnormal enlargement and dysfunction of mitochondria and causes skeletal muscle atrophy and degeneration [59]. Dulac et al. demonstrated that Drp1 knockdown in middle-aged mice leads to severe skeletal muscle atrophy, oxidative stress, and impaired autophagy, whereas Drp1 overexpression results in mild skeletal muscle atrophy and negatively affects mitochondrial quality [60]. This suggests that Drp1 content in skeletal muscle must be maintained within a certain physiological range to ensure that the rate of mitochondrial fission is neither excessive nor deficient [60].

### 2.4. Mitophagy

Mitophagy selectively removes damaged or unnecessary mitochondria through the autophagy-lysosomal system. Initiated post-mitochondrial fission, this process facilitates the encapsulation of smaller mitochondrial fragments for the selective removal of damaged mitochondria, essentially constituting a “quality control” mechanism [61]. Cells employ various mechanisms for mitophagy, including ubiquitin- and receptor-dependent pathways [62]. Several authors have proposed correlations between alterations in signaling pathways associated with autophagy and mitophagy and skeletal muscle atrophy and decreased physical function in the elderly. For instance, Picca et al. observed elevated levels of the autophagy protein p62 and the mitophagy-targeting protein BCL2/adenovirus E1B 19 kDa protein-interacting protein-3 (BNIP3) in older adults compared with younger controls, linking them to increased mitochondrial dysfunction [63]. However, Drummond et al. reported reduced expression levels of skeletal muscle mitophagy regulators BNIP3, Drp1, and Parkin in sedentary older women, attributing it to mitochondrial dysfunction [64]. Ito et al. demonstrated the crucial role of Parkin-mediated mitophagy in modulating skeletal muscle myotube atrophy by regulating mtROS production [65]. While there are some contradictory data on mitophagy, the literature results increasingly suggest a decline in mitophagy in skeletal muscle with age, which is associated with impaired skeletal muscle function. Consequently, targeting mitophagy in skeletal muscle emerges as crucial for preserving mitochondrial function and skeletal muscle mass.

## 3. Effects of Resistance Exercise Training on Aging Skeletal Muscle Mitochondria

Exercise is recognized as the most effective intervention for sarcopenia, as no specific drugs have been approved for its treatment. Evidence-based clinical practice guidelines strongly recommend physical activity as the primary treatment for sarcopenia [66]. Traditionally, AET has been recommended to improve mitochondrial adaptations [11]. However, a growing body of research suggests that RET also has a positive effect on mitochondria [67]. Here, we briefly describe the effects of RET on aging skeletal muscle mitochondria in relation to sarcopenia (Table 1).

### 3.1. Mitochondrial Reactive Oxygen Species and Mitochondrial Respiration

The effects of exercise-induced oxidative stress remain a topic of ongoing debate. The relationship between physical activity, the generation of ROS, and their potential impact is a complex and multifaceted issue. Exercise-induced ROS production can have both beneficial and detrimental effects. Moderate levels of ROS generated during exercise can promote positive physiological adaptations in active skeletal muscles. These adaptations include stimulating mitochondrial biogenesis, increasing the synthesis of antioxidant enzymes, and upregulating stress response proteins [77,78,79,80]. On the other hand, high levels of ROS production during exercise can damage macromolecular structures such as proteins, lipids, and DNA [77,78,81]. While speculation exists regarding the hormetic effect of exercise-induced ROS production in skeletal muscles, insufficient evidence suggests that extended periods of high-intensity exercise lead to tissue damage and compromised physiological functions [82,83]. Numerous studies consistently indicate that prolonged high-intensity exercise yields significant health advantages [82,84].

**Figure 1:**
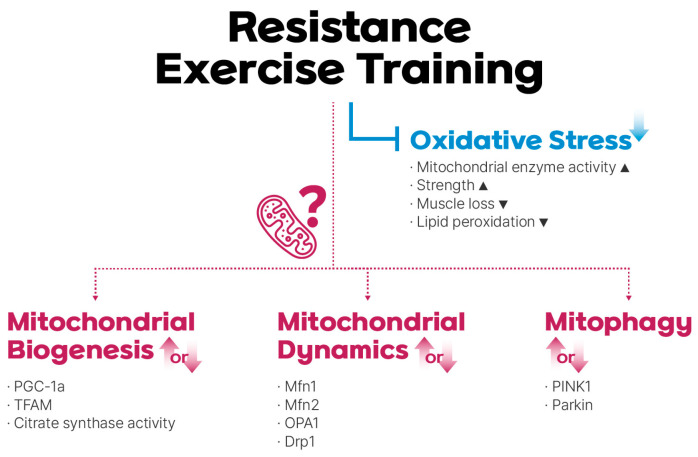
Resistance exercise training (RET)-mediated regulation of mitochondrial reactive oxidative stress (mtROS), biogenesis, dynamics, and mitophagy. RET reduces oxidative stress, although the role of mitochondrial adaptations in this process remains under debate.

The widely accepted antioxidant benefits of exercise training have led some authors to propose that exercise itself can be considered an effective antioxidant [85,86] (Figure 1). Parise et al. examined oxidative stress, antioxidant enzyme protein levels, mitochondrial enzymes, and mtDNA levels in older adults who underwent RET for 14 weeks [68]. The researchers reported that RET did not significantly alter the levels of antioxidant proteins. However, they observed a significant attenuation in DNA damage caused by oxidative stress. An analysis of mitochondrial enzyme activity revealed a significant increase in complex IV activity, suggesting a potential indirect antioxidant role for complex IV after RET. Electron leakage from the ETC can directly interact with oxygen, resulting in enhanced ROS [87]. However, efficient electron transfer at complex IV reduces ROS formation in mitochondria by ensuring complete oxygen reduction and preventing electron leaks from upstream complexes [36,88].

In a study where older adults underwent 12 weeks of moderate-intensity RET, indicators of oxidative stress were measured in blood and urine samples [89]. The authors reported that moderate-intensity RET resulted in a significant decrease in ROS production by nonenzymatic antioxidant capacity and a significant decrease in all measures of oxidative stress, including the accumulation of oxidized proteins, degree of lipid peroxidation, and oxidative DNA damage. Interestingly, the authors of this study suggested that reduced oxidative stress may be associated with increased strength and a slower rate of skeletal muscle loss.

More recently, Mesquita et al. examined the effect of 6 weeks of RET on the mRNA expression, protein levels, and enzymatic activity of several endogenous antioxidants in the skeletal muscle of 13 older males [90]. Additionally, lipid peroxidation and levels of heat-shock proteins, known for their protective role against oxidative stress, were measured. The results showed that protein levels varied, with enzymes such as catalase showing a decrease, whereas other enzymes remained unchanged. However, mRNA expression and enzymatic activity increased, and lipid peroxidation decreased. The researchers concluded that RET may be a viable approach to counteract age-related disruptions in muscle redox homeostasis through multilevel regulation of the antioxidant system.

In contrast to research suggesting that resistance exercise reduces oxidative stress, two studies found no significant improvement in mitochondrial respiratory function following RET [23,72]. Notably, these studies assessed mitochondrial respiration in skeletal muscle using high-resolution respirometry. Irving et al. investigated the effects of 8 weeks of combined exercise training (CET) compared with AET and RET on mitochondrial physiology in both young and older adults [72]. Interestingly, while all three training modalities improved muscle strength and cardiorespiratory fitness, only AET and CET led to enhanced mitochondrial respiratory function. Similarly, Robinson et al. investigated the effects of high-intensity aerobic interval training (HIIT), RET, and CET on skeletal muscle adaptations in young and older adults following a 12-week intervention [23]. This study revealed that HIIT significantly enhanced maximal mitochondrial respiration in both young and older adults, with a greater increase observed in the older group. Conversely, CET only led to a significant rise in young adults, with no improvement in the older population. RET did not elicit a statistically significant change in mitochondrial respiration for either age group. Researchers explored the potential link between changes in mitochondrial protein synthesis rate and alterations in mitochondrial respiration. Their investigation revealed no significant difference in the baseline mitochondrial protein synthesis rate between young and older participants. However, HIIT intervention led to a significant increase in the mitochondrial protein synthesis rate for both young and older groups.

### 3.2. Mitochondrial Biogenesis

Exercise is an effective stimulus for mitochondrial biogenesis, a process that involves intricate molecular pathways that promote mitochondrial growth [19,91,92,93,94]. PGC-1α is a critical regulator of mitochondrial biogenesis in skeletal muscle [95]. Exercise has been shown to increase PGC-1α expression and activity, which in turn interacts with nuclear respiratory factor 1 (Nrf1) and 2 (Nrf2) and mitochondrial transcription factor A (TFAM) to promote the transcription of mtDNA and enhance mitochondrial biogenesis [50,95]. Mitochondrial biogenesis can be measured by various methods. The first approach analyzes the expression of regulatory markers such as PGC-1α, Nrf1/2, and TFAM, using techniques such as real-time polymerase chain reaction (qPCR) or Western blotting [96]. Researchers often use the mtDNA copy number as an indicator of mitochondrial biogenesis and content, which can be quantified by qPCR [71,97]. A more accurate but complex approach involves measuring the mitochondrial content or synthesis [96]. Citrate synthase (CS) activity and the protein content of OXPHOS are commonly used as markers of mitochondrial content [98,99,100,101]. CS activity has been shown to correlate with mitochondrial volume density as measured by electron microscopy, offering a considerably faster and more practical method [100].

Although mitochondrial biogenesis that occurs in response to AET has been well studied, the effects of RET on mitochondrial adaptation remain controversial (Figure 1). Long-term (“chronic”) RET has been shown to increase the mtDNA copy number, mitochondrial contents, and PGC-1α mRNA and protein contents [70,71,72]. In a study by Ogborn et al., both young and elderly individuals underwent one-time unilateral RET [73]; the researchers observed significant increases in PGC-1α mRNA levels 3 h after exercise, followed by a rise in TFAM mRNA 24 h later in the vastus lateralis muscle. Notably, there were no age-related differences in the mitochondrial response.

It should be noted that there are conflicting findings in terms of the response of mitochondrial biogenesis and mitochondrial content to RET in older adults. Parise et al. examined the effects of unilateral RET on mitochondrial enzymes in the skeletal muscles of older adults [69]. CS activity was measured in the vastus lateralis muscle of both trained and untrained legs. Over the 12-week training period, neither the trained nor the untrained leg showed any changes in CS activity. This finding adds to the growing body of evidence suggesting no change in CS activity following RET [68,74,75]. Similarly, in three recent investigations by Flack et al., Mesquita et al., and Berg et al., the authors observed no change in the levels of mitochondrial biogenesis proteins PGC-1α, TFAM, and total OXPHOS, or mRNA expression of PGC-1α and TFAM in vastus lateralis muscle after 8–12 weeks of RET [22,74,75]. These results suggest that unchanged mitochondrial protein expression, despite greater muscle volume after RET, indicates that mitochondrial biogenesis may have occurred [75]. Moreover, decreased maximal mitochondrial respiratory rate suggests potential qualitative adaptations, such as an increased proton leak and changes in cristae morphology, rather than a simple increase in mitochondrial content [75].

### 3.3. Mitochondrial Dynamics

The effects of exercise on mitochondrial dynamics, including fusion and fission, have not been extensively studied [102]. Although it is expected that changes in cellular energy status during exercise can be achieved through mitochondrial network dynamics, the specific effects of exercise on these mitochondrial dynamic processes require further investigation [103] (Figure 1). Available evidence from a limited number of studies suggests that physical activity influences mitochondrial dynamics via multiple signaling pathways. For example, high-intensity interval training appears to induce a progressive increase in Mfn1 and Fis1 protein levels, whereas single exercise bouts result in increased mRNA expression of Mfn1 and Mfn2 at specific time points post-exercise [104,105,106]. Several methods exist to quantify protein or mRNA levels associated with mitochondrial fusion and fission. These include quantifying the levels of proteins using techniques such as Western blot analysis, immunofluorescence analysis, and quantitative immunofluorescence analysis or assessing mRNA levels using quantitative reverse transcription-polymerase chain reaction (RT-qPCR) [96].

There are relatively few investigations of mitochondrial dynamics in the skeletal muscles of older adults during RET, with most studies relying on molecular biology analyses. Recently, researchers have reported the response of mitochondrial dynamics markers following either acute or chronic RET in older adults [22]. Researchers reported no acute changes in mitochondrial dynamics-related proteins following RET. However, chronic RET (over 10 weeks) led to an increase in Mfn1, Mfn2, OPA1, and Drp1 protein levels. The authors suggest that the age-related increase in mitochondrial fusion observed might be a compensatory response to counteract the negative effects associated with aging. Age-related increases in mitochondrial fission led to fragmented networks and dysfunction [107,108]. The authors propose that fusion could act to dilute damaged mitochondrial contents by combining them with healthier mitochondria. Additionally, their data show that RET counteracts age-related dysregulation of mitochondrial dynamics, as evidenced by changes in markers of mitochondrial fusion, potentially leading to improved mitochondrial function.

In contrast, Marshall et al. did not observe significant effects after a single bout of RET on markers of mitochondrial fission/fusion dynamics [76] in a study comparing post-exercise recovery of master endurance athletes with that of untrained healthy age-matched older adults at both the 1- and 48-h time points. The researchers found that the only significant effect of RET was observed in the phosphorylation/total Drp1 ratio, a marker of mitochondrial fission, at the 1-h post-exercise time point. Importantly, this effect was observed only in the master endurance athlete group, with no changes in this or any other mitochondrial dynamics marker detected in the control group. Collectively, these findings suggest that master endurance athletes maintain more efficient mechanisms for skeletal muscle mitochondrial quality control, facilitating the fragmentation of damaged or dysfunctional organelles. Given the conflicting findings in the recent literature, the influence of RET on mitochondrial volume in older adults requires further investigation.

### 3.4. Mitophagy

The signaling pathway mediated by PTEN-induced putative kinase protein 1 (PINK1) and Parkin is recognized as an extensively investigated pathway responsible for transmitting signals related to mitochondrial damage, ultimately leading to the initiation of mitophagy [109]. Mitophagy is also partially mediated by BNIP3 and BNIP3L/NIX, which are located on the outer mitochondrial membrane [110,111]. Exercise induces mitophagy through various mechanisms, including the PINK1/Parkin pathway [112]. A recent cross-sectional study found that older individuals who engaged in regular exercise exhibited elevated levels of key proteins involved in mitophagy, specifically Parkin and BNIP3 [113]. Although some studies have suggested that exercise promotes mitophagy, the overall effect on this process during aging requires further investigation [16] (Figure 1). Studies have shown that Parkin protein levels may remain unchanged or increase at the mRNA level in elderly individuals in response to exercise [64,114]. This suggests that exercise may not strongly stimulate the PINK1/Parkin pathway in this population. However, PINK1 did not show a clear response, with some studies reporting no significant changes [114,115]. The current methods for assessing mitophagy are limited. Transmission electron microscopy (TEM) and Western blot analysis of mitochondrial protein levels are established methods for directly visualizing and quantifying mitophagy [96,116]. However, these methods lack accuracy and the ability to specifically quantify mitophagy [96,116]. In comparison, fluorescent-based strategies offer enhanced reliability and potential for live-cell imaging [96].

One limitation of the existing literature is the paucity of studies investigating the effects of RET on mitophagy in older adults. The previously mentioned study by Ogborn et al. compared the acute response of young and aged skeletal muscle mitochondria to RET [73]. They found that the levels of PINK1 and Parkin proteins in the cytosolic fraction remained unchanged after exercise, and neither age nor exercise regimen appeared to influence their interactions significantly. Given this confirmation of a mitophagy response in the mitochondria following acute RET exposure, it is critical to determine if this response persists in chronic settings [117].

A recent study by Mesquita et al. further supported these findings by demonstrating a lack of statistically significant differences in the mitophagic response of skeletal muscles in older adults following acute and chronic RET [22]. There was no change in the levels of PINK1 and Parkin proteins in the vastus lateralis tissue either 24 h after the first RET session or 72 h after 10 weeks of RET. In fact, the authors argued that the results presented do not definitively show unaltered mitophagy, as the study did not analyze the levels of phosphorylated PINK1 and Parkin proteins, which can affect their activity [118]. These results suggest that RET does not significantly affect mitophagy markers in older individuals. This finding is in contrast with the increased PINK1 protein levels observed in young skeletal muscles following chronic RET [21].

## 4. Conclusions

**Figure 2:**
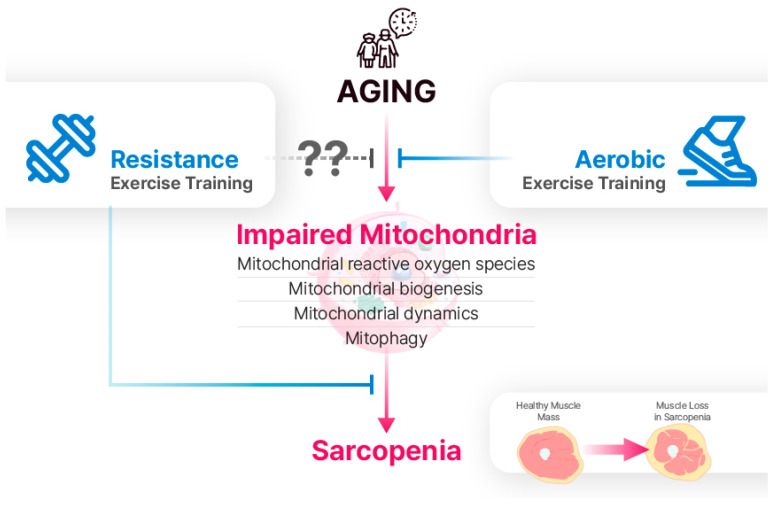
Aging leads to the impairment of mitochondrial reactive oxygen species (mtROS), biogenesis, dynamics, and mitophagy. Although the effects of RET on mitochondria in aging skeletal muscle are not fully understood, it is a well-established intervention for sarcopenia. While AET is traditionally viewed as more effective for mitochondrial adaptations, recent research suggests that RET can also have positive effects on mitochondrial function.

The findings of this review indicate that mitochondrial dysfunction contributes to the acceleration of age-related sarcopenia (Figure 2). Specifically, mtROS production, mitochondrial biogenesis, mitochondrial dynamics, and mitophagy are implicated in sarcopenia. Previous studies have demonstrated that AET reduces oxidative stress and enhances mitochondrial biogenesis and dynamics. While RET has also emerged as a promising preventive and therapeutic approach for improving muscle strength and potentially attenuating sarcopenia, much of the research on RET has focused on muscle hypertrophy and strength gains, with relatively few studies investigating mitochondrial responses to RET. Despite conflicting findings regarding the effects of RET on mitochondria in the elderly, numerous studies have reported the beneficial effects of RET on mitochondrial function in aging skeletal muscles. Further investigations are imperative to elucidate the specific mechanisms through which RET influences mitochondrial adaptation in aging skeletal muscles.

## Figures and Tables

**Table 1:** Effects of resistance exercise training on skeletal muscle mitochondria in the elderly.

Reference	Sample Size	Age	RET Protocol	RET Type	Results
Parise et al., 2005 [68]	15 Males 15 Females	68.5 ± 5.1 yr	3 times/week for 14 weeks, 50–80% of 1RM	Circuit exercise training of all major muscle group exercises	CS Activity ↔ mtDNA ↔
Parise et al., 2005 [69]	12 Males	71.2 ± 6.5 yr	Unilateral training, 3 times/week for 12 weeks, 3 sets of 10 reps at 50–80% of 1RM	Leg press Leg extension	CS Activity ↔
			Acute training, 3 sets of 10 reps using untrained leg at 80% of 1RM		CS Activity ↔
Balakrishnan et al., 2010 [70]	23 CKD patients	64 ± 10 yr	3 times/week for 12 weeks, 80% of 1RM	Chest and leg press Latissimus pull-down Knee extension Flexion pneumatic RET machines	mtDNA ↑
Sparks et al., 2013 [71]	23 Males 29 Females	57.6 ± 7.5 yr	3 times/week for 9 months, 2–3 sets	4 upper body RET 3 lower body RET Abdominal crunches Back extensions	CS Activity ↑ mtDNA ↑ protein (OXPHOS) ↑
Irving et al., 2015 [72]	5 Males 5 Females	70 yr	4–5 times/week for 8 weeks, 4 sets of 8–10 reps	Multiple muscle groups RET	mRNA (PGC-1α) ↑ mRNA (TFAM, Nrf2) ↔ mRNA (Nrf1) ↓ protein (PGC-1α) ↑ protein (TFAM, OXPHOS) ↔
Ogborn et al., 2015 [73]	9 Males	70 ± 4 yr	Acute unilateral training, 4 sets of 10 reps at 75% of 1RM	Leg press Knee extension	mt DNA ↑ mRNA (PGC-1α, TFAM, BNIP3) ↑ mRNA (Nrf2) ↓ protein (PINK1, Parkin) ↔
Flack et al., 2016 [74]	20 Males	≥60 yr	3 times/week for 12 weeks, repetitions to volitional fatigue/failure	3 upper body RET 4 lower body RET	CS activity ↔ mRNA (PGC1ɑ, TFAM) ↔
Mesquita et al., 2020 [22]	6 Males 10 Females	59 ± 4 yr	2 times/week for 10 weeks, 3 sets of 10–12 reps	Leg press Leg extensions Leg curls Barbell bench press Cable pull-downs	protein (OXPHOS, Nrf1, Mfn1/2, OPA1) ↑ protein (PGC-1α, TFAM, Drp1, Fis1, PINK1, Parkin) ↔
Berg et al., 2020 [75]	7 Males 3 Females	75 ± 9 yr	3 times/week for 8 weeks, 4 sets of 4 reps at 85–90% of 1RM	Knee extension	CS activity ↔ protein (OXPHOS) ↔
Marshall et al., 2022 [76]	7 Male	74 ± 3 yr	Acute training, 6 sets of 12 reps, 6 sets of 12 reps at 75% of 1RM	Knee extension	CS activity ↓ protein (PGC-1α, TFAM, OXPHOS, Drp1, Fis1, OPA1, Mfn2) ↔

↑: increase; ↓: decrease; ↔: no difference. 1RM: one-repetition maximum; CS: citrate synthase; CKD: chronic kidney disease; mtDNA: mitochondrial DNA; OXPHOS: oxidative phosphorylation; PGC-1α: peroxisome proliferator-activated receptor-gamma coactivator 1 alpha; TFAM: mitochondrial transcription factor A; Nrf: nuclear respiratory factor; BNIP3: BCL2/adenovirus E1B 19 kDa protein-interacting protein-3; PINK: PTEN-induced putative kinase protein; Mfn: mitofusin; OPA: optic atrophy; Drp: dynamin-related protein; Fis: mitochondrial fission.
